# Laying performance, digestibility and plasma hormones in laying hens exposed to chronic heat stress as affected by betaine, vitamin C, and/or vitamin E supplementation

**DOI:** 10.1186/s40064-016-3304-0

**Published:** 2016-09-20

**Authors:** Youssef A. Attia, Abd El-Hamid E. Abd El-Hamid, Ahmed A. Abedalla, Marfat A. Berika, Mohammed A. Al-Harthi, Osman Kucuk, Kazim Sahin, Baha M. Abou-Shehema

**Affiliations:** 1 Arid Land Agriculture Department, Faculty of Meteorology, Environment and Arid Land Agriculture, King Abdulaziz University, P.O. Box 80208, Jeddah, 21589 Saudi Arabia; 2 Animal and Poultry Production Department, Faculty of Agriculture, Damanhour University, Damanhour, Egypt; 3 Department of Poultry Nutrition, Animal Production Research Institute, Ministry of Agriculture and Land Reclamation, Agriculture Research Center, Giza, Egypt; 4 Department of Animal Nutrition, Faculty of Veterinary Medicine, Erciyes University, Kayseri, Turkey; 5 Department of Animal Nutrition, Faculty of Veterinary Medicine, Firat University, Elazig, Turkey

**Keywords:** Laying hens, Performance, Heat stress, Betaine, Vitamin C, Vitamin E

## Abstract

Heat stress had a negative effect on laying hens’ performance, thus this research was to study the influences of betaine (Bet, 1000 mg/kg betaine), vitamin C (VC, 200 mg/kg ascorbic acid), and vitamin E (VE, 150 mg/kg α-Tocopherol acetate) and their possible combinations on egg production, digestibility of nutrients, plasma hormones and reproductive organs of dual-purpose hens exposed to chronic heat stress. Two hundred and eighty eight hens and thirty-six cocks from 32 to 48 weeks of age were divided into nine treatment groups of four replicates, each containing eight hens and one cock. One group was kept under thermo-natural condition and the eight others were kept under chronic heat stress (CHS). One of these eight was used as a negative control, while the others were supplemented with VC, VE and/or betaine and their possible combinations. Body weights, laying rate, feed intake, and feed conversion ratio in hens reared under CHS rooster without any supplementation during 32 to 48 weeks of impairment (*P* = 0.0052) were recorded. Hens reared under heat stress and fed a diet supplemented with either Bet, VC, VE or combination of the supplements increased production traits. However, hens supplemented with VC showed the greatest production traits. Plasma glucose, estradiol-17 (E_2_), progesterone (P_4_), tri-iodothyronine (T_3_) and thyroxine (T_4_) decreased in hens reared under CHS and fed a diet with no supplementation compared to the other treatments (*P* = 0.001). Liver weights, spleen weights, thyroid gland weights, ovary weights, oviduct weights and oviduct lengths were lowest in hens reared under CHS and fed a diet with no supplementation (*P* = 0.0480). In conclusion, dual purpose hens reared under CHS and supplemented with VC at 200 mg/kg diet and Bet at 1000 mg/kg enhanced the laying performance and combated CHS.

## Background

In the tropic, heat stress (HS) are most important factor that negatively affects animal performance. Heat stress adversely influences feed intake, reproductive and laying performance, economical traits and decrease welfare of laying hens (Daghir 2008). High temperature negatively affect feed intake and consequently endocrine system, acid–base imbalance and organs’ functions. These adverse influences the process of egg formation at levels ovary and reproductive tract as well as process of ovulation and oviposition (Rozenboim et al. 2007; Oguntunji and Alabi 2010). Both environmental temperature and relative humidity interacted to affect HS severity (Attia et al. 2006; Tumová and Gous 2012), and laying hens strain (Franco-Jimenez et al. 2007). Vitamin C and vitamin E as antioxidant and trimethylglycine (Betaine; Bet) has been suggested to alleviate the adverse effects on HS on laying hens (Daghir 2008; Attia et al. 2009, 2011).

Betaine or Trimethylglycine and implicated in biological process such as, osmoprotective, methionine and choline sparing, fat distribution and immunity (Graham 2002). However, animal feedstuffs are poor supply for Bet and supplementation appears essential for enhancing performance and stress resistance in poultry (Wang et al. 2004). Bet has been reported to improve growth, carcass yields and muscle protein deposition in broilers (Türker et al. 2004) and in ducks (Wang et al. 2004) and reduce fat (Attia et al. 2005; Hassan et al. 2005).

Vitamin C is a water-soluble vitamin with anti-oxidant activity, protecting animals under stress (Attia et al. 2009). Vitamin C is essential for collagen, 1, 25-dihydroxy vitamin D and biosynthesis of adrenaline and corticosterones secretion, body temperature regulation and boosting immunity (Panda et al. 2008). Vitamin C supplementation was found to alleviate the negative effect of stress on metabolic process and enhances production, and boosts immunity (Daghir 2008; Attia et al. 2009; Khan et al. 2012a). Feed intake, survivability, feed intake and laying characteristics of layers were found to be enhanced by vitamin C supplementation at 200–400 mg/kg (Daghir 2008; Çiftçi et al. 2005).

Vitamin E plays a crucial role in cell-protection and free radicals scavengers (Bollengier-Lee et al. 1999; Metwally 2003; Khan et al. 2011). Catecholamines and coticosterone release and lipid peroxidation in cell membranes are initiated by heat stress. Vitamin E requirements must be supplied in order to compact heat stress. Metwally (2003) suggested that vitamin E could decrease the adverse influences of corticosterone induced by stress. Vitamin E also protects lymphocytes, macrophages and plasma cells against oxidative damage and enhances immune cell functions and proliferations (Traber 2007; Khan et al. 2012b). Hence, dietary vitamin E supplementation is essential under conditions of HS. The objective of this research was to study the influences of betaine, vitamin C and vitamin E alone or as a combination supplemented to the diet of dual-purpose hens under chronic heat stress on performance, egg yield, nutrients’ digestibility, plasma hormones and organ weights.

## Methods

The experimental protocol of this study was approved by Animal Production Research Institute Scientific and Ethical committee under registration no. 1037, code no. 01100337 approval date July 11, 2010.

### Laying hens, design, housing and diets

A total of 288 hens and 36 roosters of Mandarah strain (a native breeders classified as a dual-purpose hens). The experiment was started at 32 and terminated at 48 weeks of age. Birds were housed in an environmental-controlled lightproof house (close system—controlled for temperature, humidity and light). Chickens were divided into nine treatment groups with equal numbers in straight run experimental design. Hens were housed in 36 floor pens (2 m × 1.2 m × 2 m) furnished with wheat straw. Each treatment was replicated 4 times, each containing 8 hens and 1 cock. The birds were reared either at an optimum temperature of 22–24 °C with relative humidity (RH) of 45–55 % serving as positive control (T1) and fed with a basal diet (Table 1) or under CHS (38 °C ± 1; 55–65 % RH) for three successive days a week from 11.00 a.m. to 15.00 p.m. Optimal temperature (22–24 °C) in the hen house was maintained using cool-cell-pads. Under normal condition, the relative humidity was adjusted automatically (ranged between 45 and 55 %). The air is removed from the house by means of exhaust fans; fresh air is allowed to enter through the cool-cell-pads openings. Chickens under CHS were divided into eight groups, i.e., chickens kept under CHS and fed with a basal diet serving as negative control (T2); chickens kept under CHS and fed with a basal diet supplemented with 1000 mg/kg betaine (T3) (natural Betafin^®^ S4 contain 93 % dry Bet, Danisco Animal Nutrition, UK); chickens kept under CHS and fed with a basal diet supplemented with 200 mg/kg ascorbic acid (T4) (L-ascorbic acid; a heat stabilized product, Hoffmann-La Roche, Switzerland); chickens kept under CHS and fed with a basal diet supplemented with 150 mg/kg α-Tocopherol acetate (T5) (α-Tocopherol acetate, Hoffmann-La Roche, Switzerland); chickens kept under CHS and fed with a basal diet supplemented with 1000 mg/kg betaine and 200 mg/kg ascorbic acid (T6); chickens kept under CHS and fed with a basal diet supplemented with 1000 mg/kg betaine and 150 mg/kg α-Tocopherol acetate (T7); chickens kept under CHS and fed with a basal diet supplemented with 200 mg/kg ascorbic acid and 150 mg/kg α-Tocopherol acetate (T8) or chickens kept under CHS and fed with a basal diet supplemented with 1000 mg/kg betaine, 200 mg/kg ascorbic acid, and 150 mg/kg α-Tocopherol acetate (T9).

**Table 1:** Ingredients and composition of the experimental diet (as fed basis) fed to laying hens

Ingredients and composition	g/kg
Yellow corn, ground	663.3
Soybean meal 48 % CP	242.0
Limestone	75.0
Dicalcium phosphate	13.2
Vit + Min Premix^a^	2.5
NaCl	2.5
DL-methionine	1.5
Calculated and determined composition	
ME, MJ/kg^b^	11.62
Dry matter, g/kg^c^	907.3
CP, g/kg^c^	170.6
Ether extract, g/kg^c^	24.5
Crude fibre, g/kg^c^	39.6
Methionine, g/kg^b^	3.91
Ash, g/kg^c^	63.7
Nitrogen free extract, g/kg^c^	609.8
Meth. + Cys. (TSAA, g/kg)^b^	6.7
Lysine, g/kg^b^	8
Calcium, g/kg^b^	31
Available P, g/kg^b^	3.74

^a^Vit + Min mixture provides per kilogram of diet: vitamin A, 12,000 IU; vitamin E, 10 IU; menadione, 3 mg; Vit. D_3_, 2200 ICU; riboflavin, 10 mg; Ca pantothenate, 10 mg; nicotinic acid, 20 mg; choline chloride, 500 mg; vitamin B_12_, 10 g; vitamin B_6_, 1.5 mg; vitamin B_1_, 2.2 mg; folic acid, 1 mg; biotin, 50 g. Trace mineral (milligrams per kilogram of diet): Mn, 55; Zn, 50; Fe, 30; Cu, 10; Se, 0.10; Anti oxidant, 3 mg

^b^Calculated from NRC (1994) table values

^c^Deterimed values based on AOAC (2004)

Feeds and water were provided ad libitum throughout the experimental period. Vaccination and medical program were done according to common veterinarian care practice. Chickens were provided with 16 h light—8 h dark cycles using automatic timers to schedule the lighting regimens.

### Measurements

#### Laying performance

Laying rate (egg number/hen/d), egg weight (g), egg mass (g/hen/day) and feed intake (g/hen/d) were recorded daily for each replicate. Feed conversion ratio (FCR) was calculated as the amount of feed consumed (g) required to produce a unit (g) of egg mass (feed conversion = g feed/g egg). Feed conversion ratio was predicated every 4 weeks throughout the experimental period. Mortality rate was recorded during the experimental period, and post-mortem investigation was done by veterinarians. The data from mortality rate was utilized to estimate the survival rate.

#### Digestibility of nutrients

Four roosters at 48 weeks of age from each treatment (as four replicates of one male) were used to determine the digestibility coefficient values namely, dry matter (DM), crude protein (CP), ether extract (EE), crude fiber (CF) and nitrogen free extract (NFE) using the method described by Attia et al. (2012). Roosters were fasted for 24 h and then fed with their corresponding experimental diets for 72 h followed by collection of excreta. The excreta was collected for each replicate, cleaned from feeds and feathers, and then weighed, dried in a forced air oven at 70 °C for 36 h. Samples were then finally ground and placed in screw-top glass jars until analyses. Excreta nitrogen was measured using procedures outlined by Jakobsen et al. (1960). The proximate analysis of diet and excreta were done according to Association of official Analytical Chemists AOAC (2004). Coefficient of nutrient digestibility were calculated according to Attia et al. (2012).

#### Blood analyses

At 48 weeks of age, 5 ml blood samples were withdrawn from the brachial vein from two hens per replicate per treatment. Blood samples were collected in the morning from the overnight-fasted chickens. Each sample was collected in two tubs; one without anticoagulant, while in the other, heparin was used as an anticoagulant. Plasma and serum was obtained by centrifugation of blood at 1500×*g* for 20 min and kept at −20 °C until used for analysis. Plasma glucose (mg/dl) was measured by the method of Trinder (1969) using diagnostic kits (Diamoned Diagnstics, USA). Concentrations of plasma estradiol-17 (E_2_) and progesterone (P_4_) were assayed via radioimmunoassay (RIA) using DSL-43100 and DSL-3900, respectively (Diagnostic systems Laboratories Inc, TX, USA) according to the method described by Abraham (1977). Plasma triiodothyronine (T_3_) was analyzed using radioimmunoassay (RIA) kits (Diagnostic Systems Laboratories USA by Automatic 1275 mini Gamma Counter LKB) according to the method described by Hollander and Shenkman (1974).

##### Slaughter test

At 48 weeks of age, two hens per replicate per treatment were individually weighed and slaughtered; body organ measurements were calculated as a percentage of live body weight. Ovary, oviduct, total ovarian follicle, large yellow follicle; small white follicle were removed immediately, and then weighted separately to the nearest gram. The weights of these organs were expressed as the percentage of live weight.

##### Statistical analysis

Data were analysed using the GLM procedure of Statistical Analyses Software^®^ (SAS 1996) using one-way ANOVA according to the following model: yij = µ + τj + εij, where µ = the general mean, τj = the effect of treatment, εij = the experimental error. Before analysis, all percentages were converted to arcsine to normalize data distribution. Mean difference at *P* = 0.05 was tested using student Newman Kelus test.

## Results

As planned, the initial body weights remained similar among the treatments (*P* = 1.00) (Table 2). However, body weight changes in hens reared under CHS without any supplementation during 32–48 weeks were the lowest (*P* = 0.001). Survival rate also followed similar pattern to the body weight changes. Laying rate was greatest in hens reared under optimum thermal conditions (no heat stress); hens reared under CHS without any supplementation had lowest laying rate (*P* = 0.001). Hens reared under CHS but fed a diet supplemented with either Bet, VC, VE or combination of these supplements increased laying rate, hens supplemented with VC being greatest. Egg weights did not change among treatments (*P* = 0.325). However, egg masses were greatest in hens reared under optimum thermal conditions, and hens reared under CHS without any supplementation had lowest egg masses (*P* = 0.001). Hens reared under CHS but fed a diet supplemented with either Bet, VC, VE or combination of these supplements increased rate of laying, hens supplemented with Bet and vitamin C being greatest.

**Table 2:** Effects of dietary betaine (Bet), vitamin C (Vit. C), vitamin E (Vit. E), and their combined supplementation on some productive performance of dual-purpose hens reared under heat stress condition

Parameters	Control (+)	Heat stress treatments								SEM	*P value*
		Control (−)	Control + Bet	Control + Vit. C	Control + Vit. E	Control + Bet + Vit. C	Control + Bet + Vit. E	Control + Vit. C + Vit. E	Control + Bet + Vit. C + Vit. E		
Initial body weight, g	1573	1572	1571	1573	1574	1572	1571	1582	1569	2.393	1.00
Body weight change	198^a^	149^b^	206^a^	190^a^	206^a^	205^a^	198^a^	203^a^	197^a^	1.604	0.001
Survival rate	100^a^	98.5^b^	100^a^	100^a^	99.2^ab^	100^b^	100^b^	100^b^	100^b^	0.395	0.097
Laying rate, %	68.0^a^	60.5^f^	65.5^bc^	65.8^b^	63.6^e^	63.9^e^	65.4^bc^	64.8^cd^	64.2^de^	0.239	0.001
Egg weight, g	50.5	49.4	50.9	50.5	49.4	49.6	49.4	49.8	49.6	0.334	0.325
Egg mass, g/d/hen	34.3^a^	29.9^d^	33.3^b^	33.2^b^	31.4^c^	31.7^c^	32.3^c^	32.3^c^	31.9^c^	0.256	0.001
Feed intake, g/d	135^a^	127^h^	133^b^	131^de^	130^ef^	132^cd^	129^fg^	132^cd^	129^fg^	0.302	0.001
Feed conversion ratio	3.95^d^	4.29^a^	3.99^cd^	3.94^d^	4.16^b^	4.16^b^	4.00^cd^	4.10^bc^	4.07^bcd^	0.095	0.001

Means with at least one common superscript in a row do not differ significantly (P > 0.05)

Feed intake was greatest in hens reared under optimum thermal conditions, and hens reared under CHS without any supplementation had lowest feed intake (*P* = 0.001). Hens reared under CHS but fed a diet supplemented with either Bet, VC, VE or combination of these supplements increased feed intake, hens supplemented with VC being greatest. Hens reared under thermal conditions and hens reared under CHS and fed a diet supplemented with VC had greatest FCR compared with hens reared under CHS with or without supplementation (*P* = 0.001). Hens fed a diet with no supplementation had the worst FCR.

Nutrient digestibility of DM, EE, CF and NEF remained similar in hens reared either optimal thermal conditions or under CHS (Table 3). Digestibility of CP was greatest in hens reared at thermal conditions compared with hens reared under CHS with hens fed a diet supplemented with Bet, VC and VE together (*P* = 0.001) were intermediate. Digestibility of CP was most negatively influenced in hens reared under CHS and fed a diet with no supplementation at all.

**Table 3:** Effects of dietary betaine (Bet), vitamin C (Vit. C), vitamin E (Vit. E), and their combined supplementation on digestibility of nutrients in dual-purpose hens reared under heat stress condition

Digestibility, %	Control (+)	Heat stress treatments								SEM	*P value*
		Control (−)	Control + Bet	Control +Vit. C	Control +Vit. E	Control + Bet + Vit. C	Control + Bet + Vit. E	Control + Vit. C + Vit. E	Control + Bet + Vit. C + Vit. E		
Dry matter	69.12	68.60	69.44	69.03	69.18	69.42	69.35	68.97	69.33	0.371	0.791
Crude protein	79.60^a^	76.41^c^	78.93^ab^	78.75^ab^	78.22^b^	79.13^ab^	78.97^ab^	78.95^ab^	79.17^ab^	0.271	0.001
Ether extract	78.24	77.93	78.41	78.11	78.09	78.33	78.36	78.21	78.49	0.402	0.991
Crude fiber	24.12	23.99	24.16	23.95	24.00	24.14	24.17	24.06	24.15	0.310	0.999
Nitrogen free extract	79.34	79.23	79.45	79.32	79.40	79.56	79.50	79.52	79.42	0.355	0.999

Means with at least one common superscript in a row do not differ significantly (P > 0.05)

Plasma glucose concentrations were lowest in hens reared under CHS and fed a diet with no supplementation, and were similar all the other treatments (*P* = 0.001) (Table 4). Concentrations of serum estradiol-17 (E_2_) were lowest in hens reared under CHS and fed a diet with no supplementation, were greatest in hens reared at thermal conditions, and were similar all the other treatments (*P* = 0.001). Concentrations of serum progesterone (P_4_) were lowest in hens reared under CHS and fed a diet with no supplementation, and were greatest in hens reared at optimal thermal conditions (*P* = 0.001). Responses in plasma T_3_ and T_4_ concentrations were similar among the treatments in such a way that hens reared under CHS and fed a diet with no supplementation had lowest concentrations of T_3_ and T_4_, and hens in all other treatments were similar (*P* = 0.006). The ratio of T_3_ to T_4_ remained similar among treatments (*P* = 1.000). Although the abdominal fat was similar among treatments (*P* = 0.151), dressing percentages, liver weights, spleen weights, thyroid gland weights, ovary weights, oviduct weights and oviduct lengths were the lowest in hens reared under CHS and fed a diet with no supplementation (*P* = 0.048) (Table 5).

**Table 4:** Effects of dietary betaine (Bet), vitamin C (Vit. C), vitamin E (Vit. E), and their combined supplementation on some blood biochemical constituents in dual-purpose hens reared under heat stress condition

Parameters	Control (+)	Heat stress treatments								SEM	*P value*
		Control (−)	Control + Bet	Control + Vit. C	Control + Vit. E	Control + Bet + Vit. C	Control + Bet + Vit. E	Control + Vit. C + Vit. E	Control + Bet + Vit. C + Vit. E		
Glucose, mg/dl	227^a^	208^b^	220^a^	218^a^	217^a^	225^a^	219^a^	226^a^	225^a^	2.683	0.001
Estrogen, ng/ml	618^a^	514^c^	595^b^	590^b^	577^b^	582^b^	580^b^	586^b^	597^b^	6.213	0.001
Progesterone, pg/ml	9.16^a^	6.12^d^	9.06^ab^	7.98^c^	7.68^c^	7.74^c^	8.08^bc^	8.54^abc^	8.70^abc^	0.265	0.001
Triiodothyronine, µg/dl	0.136^a^	0.098^b^	0.126^a^	0.124^a^	0.120^a^	0.130^a^	0.132^a^	0.128^a^	0.134^a^	0.0001	0.006
Thyroxine, µg/dl	18.2^a^	13.2^b^	17.0^a^	16.8^a^	16.4^a^	17.8^a^	17.6^a^	17.2^a^	18.0^a^	0.443	0.001
Triiodothyronine/thyroxine ratio	0.0075	0.0074	0.0074	0.0074	0.0073	0.0073	0.0076	0.0074	0.0075	0.0001	1.000

Means with at least one common superscript in a row do not differ significantly (P > 0.05)

**Table 5:** Effects of dietary betaine (Bet), vitamin C (Vit. C), vitamin E (Vit. E), and their combined supplementation on carcass and some organ characteristic as relative to live body weight in dual-purpose hens reared under heat stress condition

Parameters	Control (+)	Heat stress treatments								SEM	*P value*
		Control (−)	Control + Bet	Control + Vit. C	Control + Vit. E	Control + Bet + Vit. C	Control + Bet + Vit. E	Control + Vit. C + Vit. E	Control + Bet + Vit. C + Vit. E		
Dressing, %	65.5	61.8	62.9	64.6	65.1	65.3	64.1	64.9	65.7	0.868	0.048
Abdominal fat, %	5.09	4.79	5.29	5.14	5.09	5.25	5.77	5.86	5.93	0.396	0.151
Liver weight, %	2.48^a^	1.97^e^	2.14^d^	2.25^bcd^	2.18 ^cd^	2.35^abc^	2.26^bcd^	2.43^ab^	2.40^ab^	0.044	0.001
Spleen weight, %	0.140^ab^	0.107^c^	0.124^b^	0.125^b^	0.130^b^	0.130^b^	0.128^b^	0.149^a^	0.144^ab^	0.0004	0.001
Pancreas weight, %	0.185	0.188	0.209	0.205	0.188	0.214	0.211	0.194	0.202	0.0008	0.130
Thyroid gland weight, %	0.0090^a^	0.0070^b^	0.0086^a^	0.0082^a^	0.0088^a^	0.0088^a^	0.0090^a^	0.0084^a^	0.0092^a^	0.001	0.001
Ovary weight, %	2.83^a^	2.51^d^	2.77^ab^	2.74^abc^	2.62^c^	2.62^c^	2.70^bc^	2.67^bc^	2.65^bc^	0.071	0.001
Total ovarian follicles, (n)	55.2	49.2	54.2	53.2	52.4	52.6	53.8	52.8	52.8	1.855	0.611
Large yellow follicles, (n)	5.40	4.00	5.40	5.00	5.00	5.20	5.20	5.20	5.20	0.334	0.156
Small white follicles, (n)	49.8	45.2	48.8	48.2	47.4	47.4	48.6	47.6	47.6	1.612	0.753
Large follicle weight, %	2.50^a^	2.10^b^	2.51^a^	2.50^a^	2.52^a^	2.34^ab^	2.41^ab^	2.33^ab^	2.45^ab^	0.132	0.019
Oviduct Weight, %	3.21^a^	2.40^c^	3.13^a^	3.20^a^	2.72^b^	2.74^b^	2.83^b^	2.85^b^	2.83^b^	0.105	0.001
Oviduct length, (cm)	58.3^a^	49.2^d^	56.2^ab^	58.0^a^	50.9^cd^	52.3^bcd^	53.9^abc^	54.3^abc^	54.0^abc^	1.089	0.001

Means with at least one common superscript in a row do not differ significantly (P > 0.05)

## Discussion

Layers are susceptible to heat stress due to their high metabolic heat production caused by increasing egg formation (Blem 2000). In addition, convection and radiation displayed a little heat dissipation in hens due to very effective insulation of the body surface by their feather. The lack of sweat glands and low respiratory water evaporation limit the ability to hens to maintain normal temperature during heat stress (Dawson and Whittow 2000). The optimal ambient temperature for laying hens is about 20–25 °C (Daghir 2008; Tumová and Gous 2012). Heat stress induced adverse effects on layers when temperature exceed 30 °C (Bollengier-lee et al. 1999; Attia et al. 2006), negatively influencing intake of nutrients and laying characteristics (Attia et al. 2006, 2009, 2011; Daghir 2008) as shown in the present work. In the case of the adverse influences of the heat stress of the present work, the first main issue is the survival rate. Hens reared under CHS and fed a diet with no supplementation had lower (1.5 %) survival rate and post-mortem investigation showed hens died of heat stress; exhibited a sign of heat stress such as lungs, liver and ovary haemorrhage and emphysema. High environmental temperatures causing heat stress in poultry result in stressful behavioural responses such as panting, elevated respiratory rate, and consequently dehydration, which may cause death due to heat stroke (Ayo et al. 2010). If timely intervention measures are adopted, birds may be salvaged from death, although a high rate (3.7 %) of culled birds due to heat stress has been reported during the hot-dry season in Nigeria (Ayo et al. 2010). Abd-Ellah (1995) reported 28 % increase in mortality rate in laying hens reared in arid weather conditions of Egypt. The difference in the two reported values (Ayo et al. 2010; Abd-Ellah 1995) is probably due to the greater severity of heat stress in arid conditions of Egypt, with an ambient temperature of about 43 °C.

Further evidence of negative effects of the CHS in the present work was the decreases in body weight changes (2.5 %), laying rate (11 %), egg mass (12.8 %), feed intake (5.9 %) and FCR (8.6 %) in hens reared under CHS and fed a diet with no supplementation. Decreasing feed intake is one of the major responses of laying hens in order to maintain homoeothermic and minimize heat production (Yousef 1985). The poor productive performance is mainly due to the reduction in feed consumption, which leads to less protein biosynthesis and/or less fat deposition (Yousef 1985). Plasma concentrations of T_3_ are highly correlated to feed intake and ambient temperature in birds (Yahav et al. 1995). As observed in the present study, plasma T_3_ levels drop instantly after exposure to heat stress in birds presumably in order to decrease heat production and sustain homoeothermic (Uni et al. 2001). These results reveal that the reduced feed consumption indicated in heat stressed chickens may also be as a result of the thermally-induced changes in plasma T_3_ levels. In the present study, breeders exposed to CHS significantly decreased plasma E_2_, P_4_, T_3_ and T_4_, showing impairment in the ovarian and thyroid functions.

Further evidence of the negative effect of CHS was recorded on body weight gain that showed a 24.7 % reduction in hens reared under CHS. These results correlate with the result reported by Smith (1994) who concluded that exposure to high ambient temperatures and high relative humidity altered respiration and other physiological aberrations, resulting in a reduced growth. Dale and Fuller (1980) suggested that only 63 % of growth depression in broilers due to heat stress is directly related to reduced feed intake, and concluded that under high temperature, birds try to reduce energy metabolism and protect themselves through starvation (eating less feed to satisfy energy requirement). With the starvation, fewer nutrients are available to the body, which is reflected in the reduction of body weight gain.

The present results of rate of laying are in agreement with Bollengier-Lee et al. (1999) and Mahmoud et al. (1996) who showed that heat stress reduced egg production, and this was due to an imbalance in calcium-estrogen relationship. Similarly, Christopher et al. (1995) reported a 32.7 % decrease in laying rate (82.9 vs 55.8 %) in heat-stressed laying hens compared with hens reared under optimal thermal conditions.

The negative effect of CHS on productive performance of laying hens was concurred with a significant decrease in the digestibility coefficient of CP. The reduction on CP digestibility might be due to the decrease in the activities of trypsin, chymotrypsin and amylase of broiler chickens exposed to 32 °C that may negatively affect the digestibility of amino acids (Abu-Dieyeh 2006). In addition, Dawoud (1998) indicated that the negative effect of CHS on central nervous system may reduce metabolic rate and feed consumption under CHS. In this regard, plasma glucose concentrations in hens reared CHS decreased which agreements with the depressed body weight gains and other related parameters mentioned above. Glucose is known to be a limiting factor for animal growth.

The decrease in egg production traits of hens exposed to CHS observed herein concurred with a significant decline in ovary weight percentage (11.3 %), large follicle weight (16 %), oviduct weight (25.2 %) and length (15.6 %). The ovary in poultry displays an essential role in the reproductive functions. Small white follicles (Nitta et al. 1991) produce more than 80 % of the total ovarian estrogen, which controls the reproductive tract growth and development (Campbell et al. 2003). Granulose cells of large ovarian hierarchical follicles in laying hens secreted progesterone, the major steroid hormone (Porter et al. 1991). Disorder of the ovarian function are responsible for reduced reproductive efficiency of hyperthermia hens. In literature, White Leghorn hens exposed to heat stress (42 °C) showed a significant decrease in weight of the ovarian and large follicles number compared to those reared at 24–26 °C due to the decrease in ovarian blood supply (Rozenboim et al. 2007). The increase in blood supply to the outer skin might be one of the emergent physiological responses that alleviate endogenous thermal load via vasodilatation of the skin, shank, comb and wattle and resulted in inadequate blood supply to the ovary.

Supplementing diet of hens under CHS with VC, VE and Bet partially alleviated the negative effects of the CHS. At the present work, synergetic effect existed between vitamins and Bet, thus these agents may have to be supplied in adequate amount under condition of heat stress as layers can’t syntheses adequate amount and animal feedstuffs are poor source of Bet (Wang et al. 2004 Daghir 2008; Attia et al. 2009).

For survival rate, laying rate and feed conversion rate, VC and Bet were the most effective dietary supplements for revealing the negative effect of CHS on breeder hens, and resulted in complete recovery of feed conversion ratio. The complete recovery in only feed conversion ratio indicated the decrease in maintenance energy requirements. A mixture of Bet with VE showed similar effect but did not do better than Bet or VC alone, implying that Bet or VC may be an adequate agent in hens exposed to CHS. It should be mentioned that mixture of Bet with VE was more effective than VE alone, which showed less response than Bet and VC. This mixture (Bet with VE) resulted in complete diminishing of the negative effect of CHS on FCR. This synergetic effect may indicate the different mode of action of Bet and VE. The mixture of three agents did not yield further improvement in tolerance to CHS than the single or double supplements. The results also showed that Bet with VC did not show synergetic effect, which implies similar mode of action. In addition, the positive effect of different agents on egg production traits was combined with similar increase in body weight of breeder hens, feed intake and survival rate. For feed intake, Bet was the most effective agent followed by the Bet with VC or VC with E mixture, while the least effective agent was Bet with VE and the mixture of three supplements.

It is known that feed intake was the most effective factor for egg production (Attia et al. 1995). In addition, egg production, survival rate and feed intake of layers increased due to VC supplementation at 200–400 mg/kg [Jacob (1995); Çiftçi et al. 2005; Daghir 2008). Laying rate, feed utilization for egg production, weight of egg weight and egg mass significantly increased of layers supplied with 1000 and 1200 ppm/l water (Ahmed et al. 2008)]. Betaine and VC diet showed similar effects for relief of the negative influence on growth traits of heat-stressed chickens (Farooqui et al. 2005; Attia et al. 2009).

VC is the most effective agent and its positive impact as a water-soluble vitamin can be attributed to its role as a regulator for body temperature and its antioxidant and immune enhancers (Attia et al. 2009). Synthesize of VC is adequate by adult poultry under normal condition although VC supplementation is essential under HS to relief the adverse influence of heat stress (Daghir 2008; Attia et al. 2009).

Bet showed comparable effect to VC and could replace it as an anti-stress agent without any loss in the productive traits of breeder hens. Furthermore, Bet was more effective than VE and was equal or better than the other combinations of the supplements. Similarly, 0.1 % Bet increased egg production by 10 % through stimulation the secretion in the anterior pituitary of the follicle stimulating hormone and luteinizing hormone (Zou and Feng 2002) and dietary Bet at 0.2 % improved laying rate in heat- stressed layers (Rue et al. 2002). Similarly, very low-density lipoproteins and vitellogenin the precursors of egg yolk were significantly decreased due to heat stress (Bollengier-Lee et al. 1999) and increased by Bet supplementation at 0.06 % (Lu and Zou 2006).

Indeed, fat, protein, methionine, lysine digestibility and carotenoids were enhanced in broilers challenged with coccidiosis and supplemented with Bet compared with chickens challenged and fed Bet unsupplemented diet (Augustine and Danforth 1999). In the present study, Bet improved digestibility of crude protein thus the differences in the PC was eliminated. In addition, VC and 1 g of Bet/kg diet showed similar positive effects for partial relief of the adverse influences of CHS on chickens’ performance (Attia et al. 2009). In addition, in vitro study revealed that Bet acts as an osmoprotectant in a range of bacterial groups, such as enterobacteria and lactobacilli, in avoiding dehydration in a hyperosmotic environment (Hutkins et al. 1987).

Corticosterone, catecholamines and lipid peroxidation in cell membrane increased due to exposure to HS, and animal welfare decreased; VE can lower the adverse influences of heat stress on corticosterone secretion (Metwally 2003). VE also protects lymphocytes, macrophages and plasma cells against oxidative damage and increases proliferation and cell’ functions, thus enhance the welfare of the animal. Thus, supplementation with VE is essential under condition of heat stress. VE supplementation protects cells and tissues from lipoperoxidative damage induced by free radicals (Bollengier-Lee et al. 1999; Metwally 2003). VE supplementation at 125–250 mg/kg (Kirunda et al. 2001) and 125 mg VE plus 200 mg VC/kg of diet (Çiftçi et al. 2005) improves laying rate, feed utilization, immunity and deceased the adverse effects of high ambient temperature on productivity of layers. Moreover, VE acts as a physiological synergist and as a functioning portion of specific enzymes (Franchini et al. 1991), and increases yolk precursors, vitellogenin and VLDL during exposure to heat stress, which improves yolk and egg production (Utomo et al. 1994; Bollengier-Lee et al. 1999). Correlation between circulating calcium and oestradiol are positive in laying hens (Tojo and Huston 1980) as oestradiol control 1, 25 dihydroxy cholecalciferol synthesis and the active cholecalciferol metabolite (Taylor and Drake 1984).

Although the synergetic effects of VC, E and/or Bet may enhance laying traits, it received little thoughtfulness deposited through various mode of action. For example, a combination of ascorbate and α -tocopherol impaired myoglobin oxidation, whereas α-tocopherol or ascorbate alone did not delay metmyoglobin formation (Yin et al. 1993). In addition, VC was found to boost antioxidant activity of VE and guard VE from peroxidation (Jacob 1995; Sahin et al. 2002) by restore the tocopheroxyl radicals to their active form of VE or by sparing available VE (Retsky and Frei 1995). Thus, a positive synergistic influence of 75 IU/kg of VE and 200 ppm of VC on the immune function as antibody titer increased of broilers against Brucella abortus and Newcastle modified live and dead virus vaccine was shown (Gonzalez-Vega-Aguire et al. 1995). Also, VE (125 mg/kg diet) plus VC (200 mg/kg diet) boosted laying performance in heat-stressed layers (Çiftçi et al. 2005), and VC (150 mg) and/or VE 150 mg improved laying characteristics in layers to heat stress (Ajakaiye et al. 2011). However, VE (α-Tocopherol acetate) did not affect performance of heat-stressed (33 °C) laying hens but increased serum cholesterol and immunity.

## Conclusions

The combined effect of using different antioxidants such synthetic source of VE at 150 mg/kg and C at 200 mg/kg with Bet at 1000 mg/kg as a multi-nutritional and osmoregulatory agent in the layer’ diets reared under CHS did not excess the influence of any single agent, showing that VC or Bet may be sufficient for enhancing productive traits of dual-purpose breeder hens.

## Abbreviations

AOAC: Association of official analytical chemists
Bet: betaine
CP: crude protein
CF: crude fiber
CHS: chronic heat stress
DM: dry matter
EE: ether extract
E_2_: estrogen
FCR: feed conversion ratio
HS: heat stress
NFE: nitrogen free extract
T_4_: thyroxine
T_3_: triiodothyronine
P_4_: progesterone
VC: vitamin C
VE: vitamin E
